# Systematic review and meta-analysis of the effectiveness of ECT in reducing suicidal ideation, self-harm, suicide, and mortality

**DOI:** 10.1017/S0033291725102183

**Published:** 2025-10-30

**Authors:** Hamish Naismith, Jack Wilson, Harry Costello, Neil M. Davies, Alexandra Pitman, Robert Howard

**Affiliations:** 1 Division of Psychiatry, University College London, London, UK; 2 North London NHS Foundation Trust, London, UK; 3 Department of Statistical Sciences, University College London, London, UK; 4 Department of Public Health and Nursing, Norwegian University of Science and Technology, Trondheim, Norway

**Keywords:** electroconvulsive therapy, neuromodulation, self-harm, suicide, suicidal ideation, mortality, depression, systematic review, meta-analysis

## Abstract

Suicide and self-harm in people with depression are major public health concerns; electroconvulsive therapy (ECT) is a treatment recommended in UK clinical guidelines for severe mood disorders. We aimed to investigate published literature on the effect of ECT on the incidence of suicide, self-harm, and the recorded presence of suicidal thoughts (suicide-related outcomes). We hypothesized that ECT would be associated with a reduced incidence of suicide-related outcomes and all-cause mortality. We reviewed systematically all eligible studies as specified in our protocol (PROSPERO 293393). We included studies that compared ECT against a comparator treatment, and which included suicide-related outcomes or mortality. We searched Medline, EMBASE, and PsycINFO on January 24, 2022, updated to February 12, 2025. We identified 12,313 records and, after deduplication, screened 8,281 records on title and abstract and 212 on full-text, identifying 17 eligible studies. Studies showed significant heterogeneity in methodology, outcomes, time points chosen, and study populations. Three included studies investigated change in the suicidality domain on psychological rating scales: two showed a reduction in the ECT group; the other was underpowered for this outcome. Meta-analysis of suicide outcomes showed significant statistical heterogeneity and did not detect differences in a consistent direction. Meta-analysis of other mortality outcomes showed reductions in the risk of all-cause mortality (log relative risk [logRR]: −0.29; 95% CI: −0.53, −0.05) and non-suicide mortality (logRR: −0.21; 95% CI: −0.35, −0.07). Further high-quality studies are needed, which should seek to minimize biases (particularly confounding by indication) and report a wider range of suicide-related outcomes.

## Introduction

### Background

Suicide is a major social and public health concern: globally, at least 720,000 people per year die by suicide (World Health Organization, 2025). Self-harm, defined as ‘intentional self-poisoning or injury irrespective of the apparent purpose’ (NICE, 2022b), also causes significant morbidity: an estimated 14.6 million people globally engage in self-harm each year (Knipe et al., 2022). Suicidal ideation is also a concern because it indicates severe distress and is associated with suicide (Hubers et al., 2018).

The risks of suicide are increased in patients with psychiatric disorders, especially mood disorders (Harris & Barraclough, 1997). Such risks might be reduced through effective treatments, including antidepressant medication and talking therapies (NICE, 2022a), although there is great scope to improve their effectiveness in terms of recovery (Cuijpers, Stringaris, & Wolpert, 2020). Electroconvulsive therapy (ECT) is also recommended in UK clinical guidelines for treatment of depression, mania, and catatonia when other treatments have been unsuccessful and the condition is life-threatening (NICE, 2022a, 2003).

The UK ECT Review Group (2003) found in their systematic review of randomized controlled trials (RCTs) investigating its effectiveness in treating depression that, although most of the trials were relatively old and lacked long-term follow-up, ECT was significantly more effective than pharmacotherapy in reducing symptoms of depression. However, other authors have noted that new sham-controlled RCTs were unlikely to be ethically justifiable (Kirov et al., 2021). Given its effectiveness, ECT could plausibly reduce the risk of suicide and suicidality (defined as non-suicidal self-harm, suicidal thoughts, and suicide attempts) (McManus et al., 2022). However, direct evidence for this has been considered lacking (UK ECT Review Group, 2003) and this was also confirmed in a systematic review regarding the impact of ECT on all-cause mortality (Greenhalgh et al., 2005).

Previous systematic reviews have investigated the effect of ECT on suicide-related outcomes. Chen et al. (2021) concluded that ECT reduced suicidal ideation, but noted inconsistent findings on suicide. Kucuker et al. (2021) concluded that, while earlier studies did not show a clinical effect of ECT on suicidal ideation and suicide, a majority of more recent studies did; the authors suggested this might be explained by improved study quality or refinements made to ECT techniques over time. Limitations of these previous studies, addressed in our review, include a lack of a preregistered protocol, not meta-analyzing results, and the need to update the reviews with more recent literature.

We were interested in the effect of ECT on the outcomes of suicidal ideation, self-harm, and suicide; we refer to these as suicide-related outcomes for brevity and because the UK definition of self-harm includes suicide attempt. We were also interested in the effect of ECT on non-suicide mortality, as there are also plausible mechanisms by which ECT could reduce this risk. For example, patients who are less depressed might adopt healthier behaviors and have better adherence to treatments for their physical health conditions (Rhee et al., 2021).

ECT is commonly offered as 6–12 sessions initially (NICE, 2003), administered 2–3 times per week for 2–4 weeks until maximal sustained clinical improvement is achieved; thereafter, the frequency of sessions is often tapered to minimize the risk of relapse (Espinoza & Kellner, 2022). After the acute course of ECT, antidepressant therapy is typically continued in the form of pharmacotherapy and psychological therapies. Maintenance ECT, which involves more infrequent sessions to prevent relapse, is offered on an outpatient basis to some patients (Espinoza & Kellner, 2022). Bilateral electrode placement is most commonly used, although right-unilateral placement, which causes fewer cognitive side-effects, is sometimes chosen (Kolshus, Jelovac, & McLoughlin, 2017). Refinements to the method of ECT administration over time have reduced some side effects, such as amnesia (Sackeim, 2017). Retrograde amnesia of autobiographical events is often considered the most serious adverse effect (Sackeim, 2014) and resolves more slowly than anterograde amnesia (Espinoza & Kellner, 2022). Minor side effects, including headache, jaw, and muscle pains, are common (Espinoza & Kellner, 2022).

### Aims and hypotheses

We aimed to review the literature to investigate whether ECT is associated with a reduction in the incidence of suicide-related outcomes and of all-cause mortality, and aimed to quantify any such effects.

## Methods

### Search strategy and eligibility criteria

We preregistered our review on PROSPERO (reference: 293393). We included primary, quantitative studies evaluating ECT *versus* a control (any specified treatment, including sham ECT, pharmacotherapy, placebo, or treatment as usual). We included studies in which a suicide-related outcome or mortality was a prespecified outcome measured for each participant. We had no restrictions based on age, psychiatric conditions, or physical and/or psychiatric comorbidities. Eligible study designs included, but were not limited to, RCTs, cohort studies, and case–control studies; we excluded case reports, grey literature, editorials, and opinion articles.

All authors agreed on decisions about study eligibility that arose during screening but had been unanticipated at the protocol writing stage. This included that where records were not found via Ovid, a web search was performed; if this was also unsuccessful, the record was excluded. Studies investigating only maintenance ECT were excluded for two reasons: first, maintenance ECT is often of variable duration; second, maintenance ECT generally follows an acute course of ECT treatment, and the latter could change the baseline, pretreatment level of suicidal thoughts and self-harm. We excluded psychological autopsy studies due to the recognized issue of recall bias (Johal, Appleby, & Turnbull, 2024). We excluded studies with purely naturalistic designs (due to risk of confounding by indication) and where time points were unclear or unspecified.

We searched Ovid Medline, EMBASE, and PsycINFO on January 24, 2022 and updated to February 12, 2025. We searched for ECT and suicide-related outcomes and/or mortality in titles, abstracts, and keywords using both medical subject headings and free text terms, limited to English language records. We also included any additional records referenced in the three previous systematic reviews (Chen et al., 2021; Kucuker et al., 2021; Odermatt et al., 2025) (see eSupplementary Material).

### Data extraction and analysis

We used systematic review software to identify potential duplicate records (which were manually checked before removal) and for screening: EPPI-Reviewer version 6 (Thomas et al., 2024) for the initial search, and Covidence (Veritas Health Innovation, 2024) for the update.

One author (HN) conducted all title and abstract and full-text screening, and a randomly selected 5% were double-screened by another author (JW). There was agreement about eligibility on >80% of articles, and consensus was reached after discussion with other authors (HC, RH, and AP).

Data extraction and risk of bias ratings were completed by one author (HN), with 10% of ratings checked by another author (AP). We used the Cochrane risk-of-bias tool 2 (Sterne et al., 2019) for RCTs and the Newcastle-Ottawa Scale (Wells et al., 2024) for observational studies.

We conducted meta-analyses using the *metafor* package (Viechtbauer, 2010) in RStudio version 2024.12.1 (R Core Team, 2022). Due to data heterogeneity, we conducted a random-effects meta-analysis to estimate heterogeneity variance. We solely meta-analyzed results from studies that reported numbers of outcomes of interest (and denominators) in both ECT and comparator groups and compared these using logRR. These included results from survival analyses, cohort studies with matched groups, and pseudo-populations from studies using propensity score matching.

## Results

**Figure 1.**
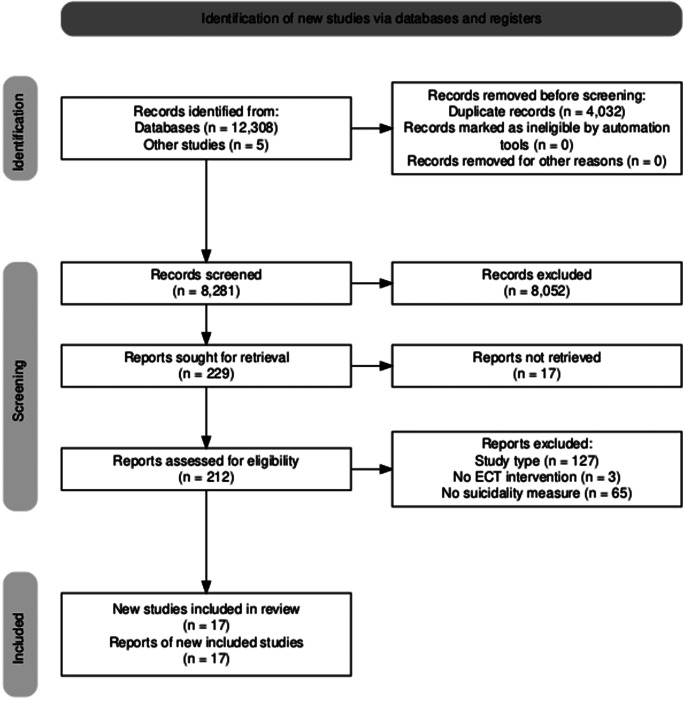
PRISMA flow diagram (Haddaway, McGuinness, & Pritchard, 2023).

We identified 12,308 records from database searches and 5 from the aforementioned three systematic reviews. We removed 4,032 duplicates and screened 8,281 records, of which 8,052 were excluded. We sought 229 reports; 17 were not retrieved. Of the 212 reports assessed for eligibility, we excluded a total of 195 for the following reasons: 127 with an ineligible study type; 3 did not include an ECT intervention; and 65 had no suicidality or mortality measure. This identified 17 final eligible studies (Figure 1).

### Change in the suicidality domain of psychological rating scales

Two RCTs and one cohort study reported suicidality as an outcome (see Table 1), which were measured on psychological rating scales (details in eSupplementary Material).

**Table 1.** Suicidal ideation

Study and country	Study design	Study population	Time points	Description of the ECT-treated/not ECT-treated group	*n*	Results statistic reported (HDRS = Hamilton Depression Rating Scale; C-SSRS = Columbia–Suicide Severity Rating Scale)	Time points
Keshtkar et al. (2011), Iran	RCT	Patients referred for ECT with refractory depression, Southwestern Iran	Pre- and posttreatment (treatment duration 3 weeks for ECT and 10 days for rTMS)	ECT	40 analyzed	Pretreatment HDRS suicidality domain 2.3 (SD 1.1). Posttreatment HDRS suicidality domain 0.3 (0.5)	Pre- and posttreatment (treatment duration 3 weeks for ECT and 10 days for rTMS)
				rTMS	33 analyzed	Pretreatment HDRS suicidality domain 1.9 (1.3). Posttreatment HDRS suicidality domain 1.4 (1.2)	
Lambourn et al. (1978), UK	RCT	Patients with depressive psychosis referred for ECT, Knowle Hospital, England	Pre- and posttreatment	ECT	16	Mean change in HDRS suicidality domain score 3.38	Pre- and posttreatment
				Sham ECT	16	Mean change in HDRS suicidality domain score 3.32	
Wang et. al (2024), China	Retrospective cohort study	Patients aged 18 years or younger, with depression or recurrent depression and suicidal ideation or a suicide attempt	Discharge from the hospital	ECT	143 matched cohort	26 patients (18.2%) had suicidal ideation at discharge, measured on C-SSRS	Discharge from the hospital
				Antidepressant therapy, matched using propensity score matching	143 matched cohort	59 patients (41.3%) had suicidal ideation at discharge, measured on C-SSRS	

Keshtkar, Ghanizadeh, and Firoozabadi (2011) conducted an RCT that analyzed patients referred for ECT with refractory depression in Iran. Participants were randomly assigned to either ECT or repetitive transcranial magnetic stimulation, but were unblinded. They compared the mean suicidality score pre- and postintervention. They analyzed 73 participants, and found both interventions significantly decreased suicidality, but there was a greater reduction in the ECT group.

Lambourn and Gill (1978) conducted an RCT that included fewer participants (32) and in which ECT was compared to sham ECT. They did not find a significant difference in mean change in the suicidality domain scores between the ECT and sham ECT groups.

Wang et al. (2024) conducted a retrospective cohort study of adolescent inpatients in China with major depression, all of whom had clear suicidal ideation or suicide attempt. They compared patients who received ECT to patients who received antidepressant pharmacotherapy. They used propensity score matching: differences between groups were balanced, except for a longer duration of hospital stay in the ECT group. They found that significantly fewer patients treated with ECT had suicidal ideation at discharge.

### Self-harm

Three included studies reported self-harm as an outcome. Jørgensen, Rozing, Kellner, and Osler (2020) studied patients with a first hospital contact due to depression or recurrent depression. Their outcome included patients with discharge diagnoses of poisoning and intentional self-harm. They found that patients with mild depression had the highest risk of self-harm (adjusted hazard ratio [aHR]: 2.69, 95% confidence interval [CI]: 1.65–4.50), followed by moderate, severe, and then severe depression with psychotic features (aHR: 1.40, 95% CI: 0.99–1.99).

Hedna et al. (2024) studied adults aged ≥75 with a diagnosis of moderate or severe depression recorded as the main cause of hospitalization. They compared patients who received ECT to controls who received no ECT during the 6-year study period, matched using exact and propensity score matching. They did not find a significant difference between groups in suicidal behavior (a composite outcome defined as suicide and nonfatal self-harm).

Salagre, Rohde, and Ostergaard (2021) conducted a mirror-image study that analyzed the incidence of self-harm and suicide attempts pre- and post-ECT in patients with unipolar and bipolar depression, psychotic disorder, and personality disorder. They found statistically significant reductions in the incidence of self-harm/suicide attempts in the 1- and 3-month post-ECT periods across all diagnoses (for patients with depression and psychosis, reductions at later periods were also seen). Comparable mirror-image analyses in several matched control groups found a reduction in some groups, but effect sizes were smaller compared to the ECT analyses.

### Suicide and other causes of mortality

Thirteen studies reported mortality, with some reporting multiple outcomes: eight reported all-cause mortality, nine reported suicide mortality, three reported nonsuicide mortality, and one reported a composite outcome of suicide and nonfatal self-harm (see Table 2).

**Table 2.** Observational studies reporting self-harm and mortality

Study and country	Study design	Study base/cohort	Description of ECT-exposed/ECT-unexposed populations	*n*	Results statistic reported ECT vs. comparator (OR = odds ratio; RR = relative risk; HR = hazard ratio; a = adjusted; pw = propensity-weighted; NS = not significant; NR = not reported)				Time points and observation periods	Other time points or subgroup results reported
					All-cause mortality	Suicide mortality	Nonsuicide mortality	Self-harm		
Ahmadi et al. (2016), USA	Nested case–control study using data from Veterans Health Administration (VHA) electronic medical records from the Captain James Lovell Federal Health Care Center in North Chicago.	Veterans with depression and comorbid PTSD at James Lovell Federal Health Care Center.	Patients with depression and comorbid PTSD treated with bifrontal ECT.	92	aRR: 0.54 (95% CI: 0.24–0.86)	aRR: 0.36 (95% CI: 0.10–0.45), estimate calculated in a sub-study using a bootstrapping technique (*n* = 2,634)	NR	NR	8 years follow-up (study reports this as both mean and median, but the endpoint is reported as 8 years for all patients).	
			Age, gender, and enrolment-time matched controls who were also under the care of the Captain James Lovell Federal Health Care Center and had depression with comorbid PTSD. Treatments they received were not specified, but included antidepressants at baseline.	3,393 (*n* = 2,634 in a sub-study of suicide using a bootstrapping technique)						
Babigian and Guttmacher (1984), USA	Psychiatric case register-based cohort study	Patients attending Monroe County Hospital. No specific criteria regarding diagnostic group or treatment, but restricted to patients with depression for analysis.	Received first ECT.	1,173 in the total sample	5-year RR: 0.7 (NS, no CI reported and not able to construct from available data)	5-year RR: 0.94 (NS, no CI reported and not able to construct from available data)	NR	NR	January 1, 1961, and December 31, 1975, subdivided into three 5-year groupings for time trend analysis.	RR death: 0.9 and RR suicide: 1.17 from ‘registry’, which is derived from census estimates.
			First lifetime psychiatric hospitalizations during the study period (this group included a majority of patients who did not receive ECT, but some who did, with the authors noting the majority of patients who received their first ECT received it during a first hospitalization).	21,227 in the total sample						
Hedna et al. (2024), Sweden	Registry-based cohort study	Swedish residents aged ≥75 years with a diagnosis of moderate or severe depression recorded as the main cause of hospitalization.	At least one ECT session as an inpatient during the study period	1,802	Multivariate aOR: 0.35 (95% CI: 0.23–0.52) for ≤3 months	Univariable OR: 0.73 (0.44–1.23) for ≤3 months for suicidal behavior (composite outcome of suicide and nonfatal self-harm).	NR	Univariable OR: 0.73 (0.44–1.23) for ≤3 months for suicidal behavior (composite outcome of suicide and nonfatal self-harm).	Persons were followed for 1 year after discharge from the index hospitalization	Multivariate aOR: 0.65 (95% CI: 0.50–0.83) for 4 months–1 year
			Received no ECT during the study period, matched using exact and propensity score matching.	4,457						
Jørgensen et al. (2020), Denmark	Registry-based cohort study	First-time hospital contact due to a single episode or recurrent depression.	Recorded on the registry as having received ECT.	5,004	aHR: 0.92 (0.71–1.14) mild depression; aHR: 0.83 (0.73–0.95) moderate; aHR: 0.96 (0.85–1.09) severe; aHR: 0.70 (0.58–0.82) severe + psychosis	aHR: 6.99 (3.30–14.43) mild depression; aHR: 3.37 (2.31–4.91) moderate; aHR: 2.84 (1.95–4.15) severe; aHR: 1.10 (0.55–2.20) severe + psychosis	NR	aHR: 2.69 (1.65–4.50) mild depression; aHR: 2.42 (1.95–3.00) moderate; aHR: 2.06 (1.65–2.56) severe; aHR: 1.40 (0.99–1.99) severe + psychosis	Reported as 0–1 year, 1–11.3 years, and for the total duration of the observation period (up to 11.3 years). January 1, 2005 to December 31, 2015.	
			Not recorded as having received ECT. No other restrictions on the comparison group, but different models adjusted for age, sociodemographic factors, and depression severity.	87,891						
Kaster et al. (2022), Canada	Retrospective cohort study using data from all designated psychiatric inpatient units in Ontario, Canada, using linked population-based administrative healthcare databases.	Admitted to hospital for >3 days and diagnosis of unipolar or bipolar depression.	Recorded from the physician billing code as having received ECT.	4,982	pwHR: 0.75 [0.58–0.97]	pwHR: 0.53 [95% CI 0.31–0.92]	pwHR: 0.83 [0.61–1.12]	NR	Within 1 year of discharge from the index hospitalization.	csHR suicide at 90 days 0.39 (95% CI: 0.16–0.91), at 180 days 0.55 (0·27–1.11)
			Not recorded as having received ECT (patients could contribute to the non-ECT group if their discharge from an admission that included ECT was >365 days ago). No restrictions on treatment received, but propensity matched on variables including sociodemographic, clinical, and treatment variables.	62,345						
Liang et al. (2017), Taiwan	Case–control study using inpatient data from the National Health Insurance Research Database (NHIRD), derived from the claims data of the National Health Insurance (NHI) program of Taiwan	Inpatients with psychiatric disorders	Recorded as having received ECT.	1,571	aOR in-hospital mortality: 1.409 (95% CI: 0.897–2.215, *p* 0.137)	NR	NR	NR	Mortality occurring during admission during the study period (1997–2003)	
			Inpatients not recorded as having received ECT, no other restrictions on the comparison group. Analysis was adjusted for patient characteristics and other confounders (not fully specified).	827,328						
Liang et al. (2018), Taiwan	Cohort study using data from NHIRD	Inpatients with unipolar disorder or bipolar depression aged >10 years with recorded gender in the database.	Patients with unipolar disorder or bipolar disorder who received ECT	487	NR	aHR: 0.803 (95% CI: 0.621–0.938, *p* = 0.044)	NR	NR	January 1, 2000, to December 31, 2013. Median follow-up time 4.4 years for the ECT group and 2.76 years for the non-ECT group.	Different stratum-specific adjusted HRs: 0.79 for unipolar depression (*P* = .041); 0.923 for bipolar disorder (*P* = .254).
			Inpatients with psychopharmacotherapy with ≥3 admissions were randomly matched (ratio, 1:4) by age, sex, and diagnosis.	1,948						
Munk-Olsen (2007), Denmark	Register-based cohort study.	Patients with at least one admission to the psychiatric hospital in Aarhus, Denmark. Included patients with depression, bipolar, and psychotic disorders, including schizophrenia.	Recorded on the registry as having received ECT.	783 ECT recipients died during the study period	aRR: 0.82 (95% CI: 0.74–0.90)	aRR: 1.20, (95% CI: 0.99–1.47)	NR	NR	All inpatients in the hospital from 1976 to 2000 (up to 24 years). Reports RR stratified by days since discharge (1–7; 8–30; >30) and ECT recency (within 7 days; 1–4wks; >4wks)	aRR: 4.82 (95% CI: 2.12–10.95) within 7 days of ECT; aRR: 1.48 (0.69–3.16) within 1–4wks; aRR: 1.23 (1.01–1.52) >4wks
			Not recorded as having received ECT. No other details reported, but groups adjusted for age, gender, diagnosis, calendar period, and age.	5,781 other psychiatric inpatients died during the study period						
Osler et al. (2022), Denmark	Registry-based cohort study.	Danish citizens with a first-time hospital contact due to an affective disorder	ECT	6,943	NR	NR	aHR: 0–30 days after first ECT: 0.28 (95% CI: 0.13–0.61) in patients without comorbidities and 0.25 (0.13–0.45) in patients with comorbidities	NR	0–30 days; 30–365 days; 365–5196 days; 0–5196 days.	aHR: 30–365 days 0.39 (95% CI: 0.28–0.53) in patients without comorbidities and 0.59 (0.43–0.71) in patients with comorbidities. Results were also reported for later timepoints.
			Not ECT-treated. Results were adjusted for sex, age, education, marital status, diagnostic subtype, number of chronic somatic diseases (and psychopharmacological treatment for deaths from natural causes)	167,552						
Peltzman et al. (2020), USA	Case–control study using data from the VHA electronic medical record data.	Veterans receiving mental healthcare, not restricted by diagnosis.	Received ECT	14,810	NR	OR = 1.31 (95% CI: 0.94–1.96, *P* = 0.095)	NR	NR	Within 1 year following ECT	
			Did not receive ECT in the year analyzed (patients could move from the ECT to the non-ECT groups if, after 2 years, they had not received further ECT). Controls not restricted to any specific treatment, but matched on sociodemographic, clinical, and treatment factors, including psychotropics.	58,369						
Rhee et al. (2021), USA	Cohort study of Medicare-insured patients using data from the Medicare fee-for-service claims database and the National Death Index.	Medicare-insured psychiatric inpatients aged ≥65 years	One or more ECT treatments.	10,460	aHR: 0.61, (95% CI: 0.56–0.66)	HR: 0.44 (95% CI: 0.21–0.91) at 1 month; HR: 0.52 (0.29–0.92) at 2 months; HR: 0.56, (0.37–0.92) 3 months; HR: 0.87 (0.59–1.28) at 6 months; HR: 0.92 (0.68–1.25) at 12 months.	NR	NR		
			Psychiatric inpatients who did not receive ECT, matched on age, gender, principal hospital diagnosis, past-year psychiatric hospitalizations, past-year suicide attempts, and Elixhauser comorbidity index.	31,160						
Rönnqvist et al. (2021), Sweden	Register-based cohort study.	Patients with any record of inpatient care for depression (moderate, severe, or severe with psychosis).	Received ECT (unilateral for 4,781, bilateral for 539 patients, 205 unknown)	5,525	NR	HR: 0.72 (95% CI: 0.52–0.99)	NR	NR	Within 12 months of admission. Study period: January 1, 2012, to October 31, 2018. Within 3 months of admission were also reported.	Psychotic features HR: 0.20; 95% CI: 0.08–0.54 45–64 yrs HR: 0.54; 95% CI: 0.30–0.99 ≥ 65 yrs HR: 0.30; 95% CI: 0.15–0.59 ≤ 44 yrs HR: 1.22; 95% CI: 0.68–2.16
			Patients on the registry with depression were not recorded as having received ECT. Controls are not restricted to any specific treatment, but matched on sociodemographic, clinical, and treatment factors, including antidepressant use.	5,525						
Salagre et al. (2021), Denmark	Register-based mirror image study.	Patients with diagnoses of depression, bipolar disorder, psychotic disorder, or personality disorder	Patients who received ECT	10,986	NR	NR	NR	Statistically significant reductions at 1-month post-ECT periods across all diagnoses (83% depression, 72% bipolar, 82% psychotic disorder, 83% personality disorder) and at 3-months	1-, 3-, and 6-month, and 1- and 2-year mirror image analyses (indexed to date of first ECT session or admission date for some of the comparator groups)	Significant reductions at 6 months (psychotic disorder, depression, and bipolar disorder) and 1 year (depression and bipolar disorder)
			Patients matched on age, sex, and diagnostic category	73,719				No statistically significant changes, except for in patients with unipolar depression (24% decrease at 1 year and 33% decrease at 2 years)		
Watts et al. (2021), USA	Cohort study of Department of Veterans Affairs patients using electronic medical records from the Department of Veterans Affairs healthcare system	Department of Veterans Affairs patients	Department of Veterans Affairs patients who received ECT during 2000–2017 (inpatients and outpatients)	123,479 individual ECT treatments provided to 8,720 patients (including 5,157 initial index courses of ECT)	OR all-cause mortality in the year after their index course of ECT (of discharge in controls) 0.87 (95% CI: 0.79–1.11)	NR	OR nonsuicide mortality 0.79 (95% CI 0.66–0.95)	NR		30-day mortality OR all-cause 1.06 (95% CI 0.65–1.73) and OR nonsuicide mortality 1.02 (0.58–1.8)
			Matched group of mental health inpatient discharges who did not receive ECT between 2010 and 2017, excluding patients whose inpatient admission was due to a primary substance misuse disorder diagnosis.	486,214						

Babigian and Guttmacher (1984) was a cohort study using a US psychiatric hospital register. This compared patients who received ECT to patients with a first hospitalization. Systematic differences were noted between groups: ECT recipients had a longer median length of stay and were significantly more likely to have depression and to be older. As a result, the authors restricted some of their analyses, including those for mortality, to patients with depression. They calculated mortality rates from the registry by dividing the number of deaths in each age group by the number of years at risk. A second analysis was conducted by following patients for 5-year periods (regardless of subsequent outcomes). They found no significant differences in the outcomes of suicide and all-cause mortality, apart from one demographic group: all adults aged ≥75, and women in this age group treated with ECT had lower all-cause mortality than the non-ECT group. They reported similar mortality rates for suicide between the ECT and non-ECT groups, but accidental and circulatory deaths were significantly lower in the ECT group.

Liang, Chung, Tsai, and Chien (2017) conducted a cohort study using data from the Taiwan National Health Insurance Research Database (NHIRD). They found ECT was not a statistically significant predictor of in-hospital mortality.

Liang et al. (2018), also using the NHIRD, investigated the risk of suicide in inpatients with unipolar or bipolar depression. Their control group was patients who received pharmacotherapy, matched on age, sex, and diagnosis, and, to ensure similar illness severity, those who had had at least three psychiatric hospitalizations. They reported a 19.7% lower risk of suicide in ECT recipients compared to controls: aHR: 0.803, 95% CI: 0.621–0.938. There were significant associations between reduced mortality and unipolar depression (aHR: 0.79, 95% CI: 0.597–0.946) but not for bipolar disorder overall. Further analyses stratified by affective state in bipolar disorder showed a significant reduction in suicide mortality for bipolar depression (aHR: 0.805; 95% CI: 0.514–0.987), but no significant associations for mania and mixed affective states.

As described above, Jørgensen et al. (2020) studied patients with a first hospital contact due to depression. They found a decreased risk of mortality in the ECT group. In analyses stratified by depression severity, in the group with severe depression with psychotic features, the aHR for all-cause mortality was lower for patients who received ECT compared to those who did not (aHR: 0.70, 95% CI: 0.58–0.82). In the group with mild depression, the aHR for patients who received ECT versus those who did not was 0.92 (95% CI: 0.71–1.14). For the outcome of suicide, in patients with mild depression, the risk of suicide was higher in patients who received ECT compared to patients who did not (aHR: 6.99, 95% CI: 3.30–14.43). The risk was also elevated in patients who received ECT in stratified analyses for moderate and severe depression and depression with psychotic features (aHR: 1.10, 95% CI: 0.55–2.20).

Rönnqvist, Nilsson, and Nordenskjöld (2021) conducted a cohort study of patients with any record of inpatient care for moderate-to-severe depression. Their outcome was suicide, the definition of which included deaths following events of undetermined intent. Overall, there was a significantly decreased risk of suicide within 12 months of discharge in patients who received ECT compared to the non-ECT group (HR: 0.72, 95% CI: 0.52–0.99). In analyses stratified by severity, only the severe depression group result was significant: the risk of suicide was reduced in the 3 months following admission (aHR: 0.20, 95% CI: 0.08–0.54). ECT was significantly associated with reduced suicide risk in patients aged 45–64 and >65 years but not in patients aged <45 years.

Kaster et al. (2022) conducted a cohort study of patients with unipolar or bipolar depression admitted for more than 3 days. Patients who received ECT were compared to patients unexposed to ECT in that calendar year. They used propensity score matching on over 100 covariates. They reported a decreased risk of suicide mortality (HR: 0.53, 95% CI: 0.31–0.92) and all-cause mortality (HR: 0.75, 95% CI: 0.58–0.97) but not nonsuicide mortality at 1 year following discharge. In an additional analysis, they analyzed the number of inpatient ECT treatments received (see Supplementary Results).

Munk-Olsen et al. (2007) conducted a Danish registry cohort study, which included patients with depressive and psychotic disorders. Patients who received ECT had a reduced relative risk of death from natural causes: adjusted relative risk (aRR): 0.82 (95% CI: 0.74–0.90). The risks of death by unnatural causes, including suicide, were not significantly different. They further analyzed the risk of suicide at two time points. First, in the analysis according to days since discharge, they used patients discharged 30 days ago as the reference and found all other groups had an elevated risk (highest in patients discharged within the past 7 days, aRR: 9.49, 95% CI: 6.80–13.24). Second, in the analysis by recency of ECT treatment, the reference group was all patients who received no ECT treatment (regardless of admission status/recency of discharge); risk was elevated in two of the three ECT groups: patients who received ECT within the past 7 days (aRR: 4.82, 95% CI: 2.12–10.95) and over 4 weeks ago (aRR: 1.23, 95% CI: 1.01–1.52).

Osler, Rozing, Jorgensen, and Jorgensen (2022) studied mortality in patients with and without comorbidities. They found a lower mortality risk in ECT-treated patients compared to non-ECT-treated patients, regardless of whether they had a comorbidity (aHR: 0.59, 95% CI: 0.43–0.71) or not (aHR: 0.39, 95% CI: 0.28–0.53). They reported an increased risk of unnatural deaths (including suicide): the risk at 30–365 days following ECT was increased in patients with comorbidities (aHR: 2.56, 95% CI: 1.80–3.62) and those without (aHR: 2.49, 95% CI: 1.84–3.35). They interpreted this as likely due to confounding by indication, because the increased risk effect extended well beyond the acute treatment course (0–5,196 days following ECT).

Two studies had an older adult sample. Rhee et al. (2021) studied US Medicare-insured adults aged ≥65 years. They analyzed suicide and all-cause mortality at different time points following hospitalization. The ECT group had a significantly lower risk of all-cause mortality at all time points up to 1 year (1-year aHR: 0.61, 95% CI: 0.56–0.66). For suicide, there was a reduced risk at up to 90 days (aHR: 0.56, 95% CI: 0.34–0.92), but not thereafter. They also found that patients who received subtherapeutic ECT (defined as <5 treatments within the first 30 days of treatment) had a similar survival trajectory to non-ECT controls. The authors suggested their findings constituted evidence against the hypothesis that lower mortality in ECT recipients could reflect selection bias (i.e. that more medically unwell patients are not selected for ECT).

As described under the self-harm category, Hedna et al. (2024) studied depressed adults aged ≥75. They did not replicate the finding in Rhee et al. of reduced risk of suicide, but they used a composite outcome of suicide and nonfatal self-harm. Their result was not incompatible with a short-term reduction in suicide risk: at ≤3 months, the univariable odds ratio (OR) for suicidal behavior was 0.73 (95% CI: 0.44–1.23). For all-cause mortality, they found a significant reduction in favor of ECT at ≤3 months following hospital discharge: multivariable OR: 0.32 (95% CI: 0.22–0.48). The risk was also reduced from 4 months to 1 year post-discharge: OR: 0.65 (95% CI: 0.50–0.83).

Three studies in this outcome category studied veteran samples. Peltzman, Shiner, and Watts (2020) was a case–control study of Veterans’ Health Administration mental health service users. The exposure was defined as receipt of ECT in the index year or the year prior. On the unmatched analysis, there were significant differences between the two groups on demographic characteristics, prevalence of psychiatric diagnoses, and suicide risk (elevated in the ECT group). After propensity score matching, OR of suicide death in the year after ECT was 1.56 (95% CI: 1.11–2.18), but this was no longer significant in the adjusted model (OR: 1.31, 95% CI: 0.94–1.96).

Ahmadi et al. (2016) conducted a nested case–control study of patients with depression and comorbid post-traumatic stress disorder. They reported an estimated 46% reduction in risk of all-cause mortality (RR: 0.54, 95% CI: 0.24–0.86), and a 64% reduction in risk of suicide in patients receiving ECT compared to a matched non-ECT group (RR: 0.36, 95% CI: 0.10–0.45).

Watts, Peltzman, and Shiner (2021) conducted a cohort study, which included outpatients receiving ECT, and used as a comparator group patients who were discharged following hospitalization (excluding those whose primary reason for admission was a substance misuse disorder). Outcomes were assessed at 30 days and 1 year, indexed from the first treatment date for the ECT group and the discharge date for the inpatient group. The groups were significantly different in terms of demographics, prevalence of diagnoses, and service use before propensity score matching. Compared to the matched group, the ECT group had a lower 1-year risk of nonsuicide mortality (OR: 0.79, 95% CI: 0.66–0.95); the result for all-cause mortality was 0.87 (95% CI: 0.79–1.11).

### Risk of bias

Most observational studies were of good quality, and many used registry data (Tables 3 and 4). Liang et al. (2017) scored poorly on comparability: details of adjustment for patient characteristics were unclear, and follow-up was only until hospital discharge. Three studies were of fair quality: one mirror-image study (Salagre et al., 2021) and two case–control studies in which the sample was veterans, which are not representative of the typical population receiving ECT (Ahmadi et al., 2016; Peltzman et al., 2020).

**Table 3.** Observational studies were rated using the Newcastle-Ottawa rating scale

Study	Selection	Comparability	Exposure	Overall quality
Ahmadi et al. (2016), USA	★★	★★	★★★	Fair
Babigian and Guttmacher (1984)	★★★★	★★	★★	Good
Hedna et al. (2024)	★★★★	★★	★★★	Good
Jørgensen et al. (2020)	★★★★	★★	★★	Good
Kaster et al. (2022)	★★★★	★★	★★	Good
Liang et al. (2017)	★★★		★	Poor
Liang et al. (2018)	★★★	★★	★★	Good
Munk-Olsen (2007)	★★★	★★	★★★	Good
Osler et al. (2022)	★★★★	★★	★★★	Good
Peltzman et. al (2020)	★★	★★	★★★	Fair
Rhee et al. (2021)	★★★★	★★	★★★	Good
Rönnqvist et al. (2021)	★★★★	★★	★★★	Good
Salagre et al. (2021)	★★	★	★★	Fair
Wang et. al (2024)	★★★	★★	★★★	Good
Watts et al. (2021)	★★★	★★	★★	Good

**Table 4.**
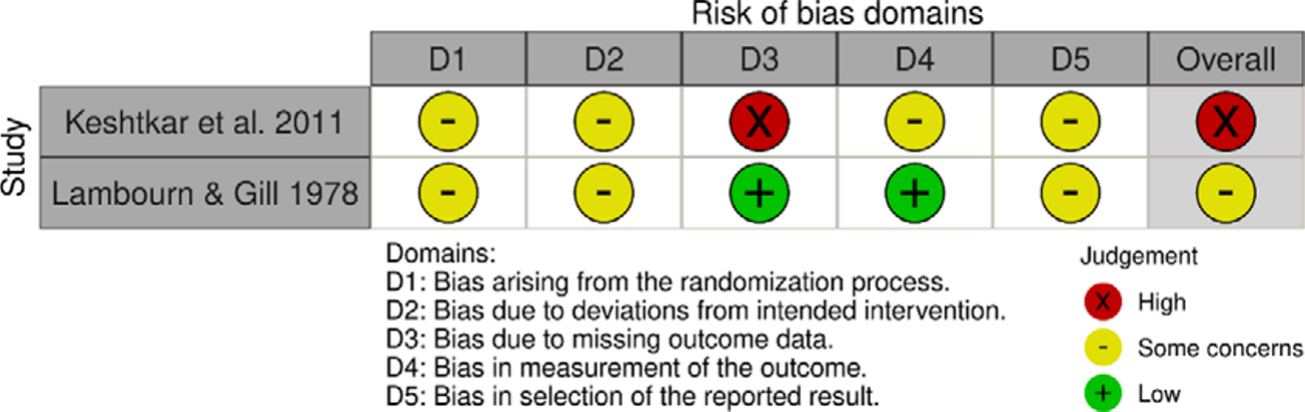
RCTs rated using RoB2 (McGuinness & Higgins, 2021)

Keshtkar et al. (2011) was considered at high risk of bias due to participants being unblinded to the interventions. There were some concerns about bias in Lambourn et al. (1978) in three domains, but the participants were blinded.

### Meta-analysis

We could not include each study from Table 1 in our meta-analyses; not every study reported the number of events for all three outcomes (all-cause, suicide, and nonsuicide mortality), and we excluded studies where the denominator was not reported or was unclear (as for Babigian & Guttmacher, 1984).

**Figure 2.**
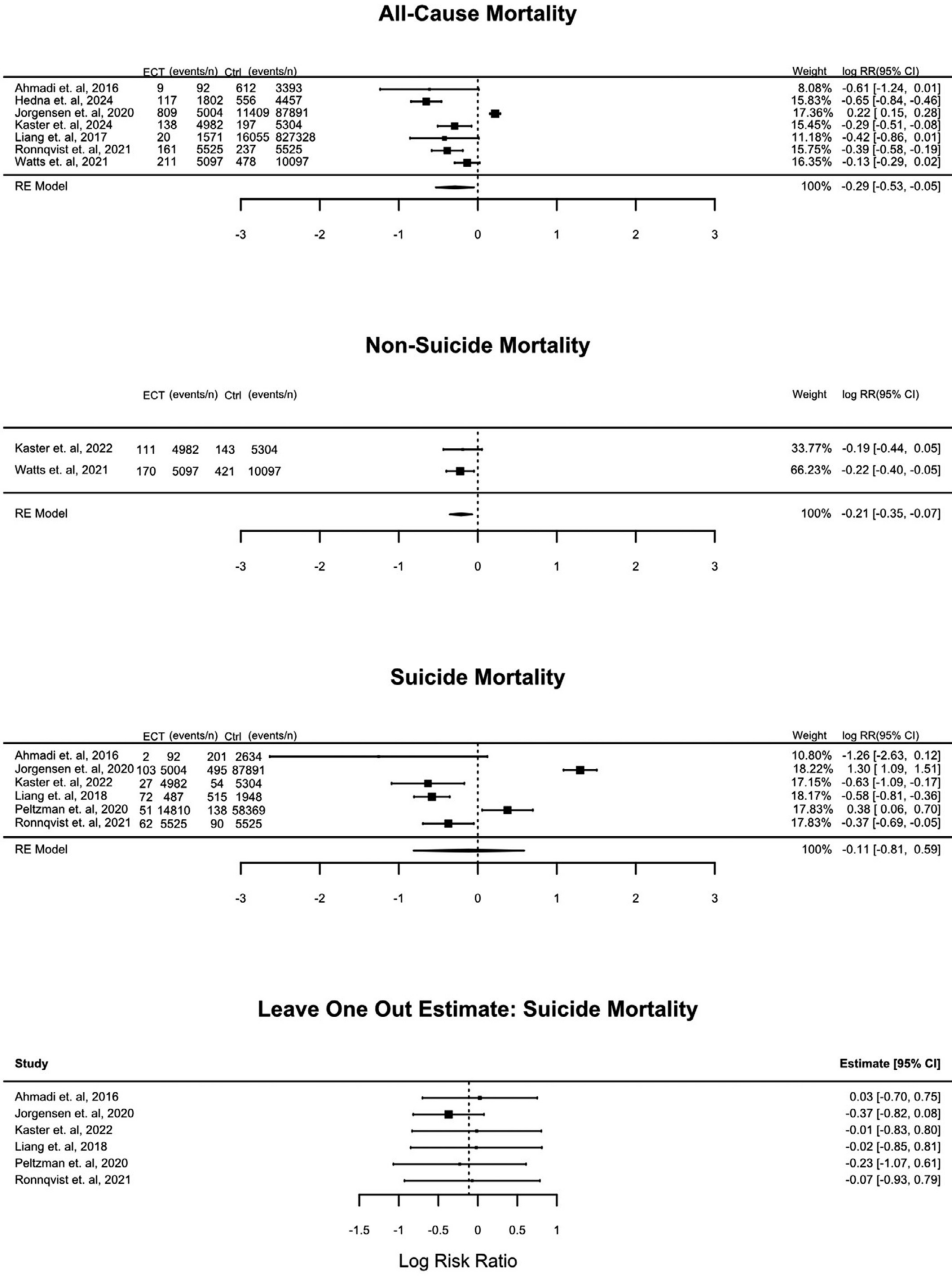
Forest plots of the effect of ECT on all-cause, nonsuicide, and suicide mortality (including leave one out analysis).

Meta-analyses of all-cause mortality (seven studies), nonsuicide mortality (two studies), and suicide (six studies) are presented in Figure 2. We included outcomes at 12 months if these were reported; if not, we used results for the entire study period: 9 years in Ahmadi et al. (2016), 11.3 years in Jørgensen et al. (2020), and 13 years in Liang et al. (2018). We also included studies that reported unadjusted results (Jørgensen et al., 2020) and results from groups that were balanced by propensity score matching (Kaster et al., 2022; Rönnqvist et al., 2021). It should also be noted that cohort studies and survival analyses deal with censoring differently.

The forest plot for suicide showed high statistical heterogeneity (*I* ^2^ = 96.39%) and did not show differences in a consistent direction of effect for ECT or comparator treatments across all studies. We conducted a leave-one-out estimate, which showed that heterogeneity would remain high regardless of which study was excluded, and that the result would still neither favor ECT nor comparator treatments.

Meta-analysis of all-cause mortality and nonsuicide mortality was in favor of ECT, with significant heterogeneity in the all-cause mortality results (*I* ^2^ = 91.43%).

## Discussion

### Main findings

We found in our systematic review that of the three included studies that investigated the outcome of suicidal ideation, two showed a significant reduction (Keshtkar et al., 2011; Wang et al., 2024).

Three included studies had self-harm as an outcome and reported conflicting results, which could be due to the differing definitions of self-harm and the clinical populations included.

The high statistical heterogeneity in our meta-analyses likely reflects the fact that, due to our broad inclusion criteria, the observational studies identified for our review employed a variety of study designs, including case–control, nested case–control, and cohort designs, which often utilized registry data. As a result, a range of outcome measures were presented, including RR, HR, and OR.

Our meta-analyses showed ECT was associated with a reduction in all-cause and nonsuicide mortality, despite substantial heterogeneity. This finding could be attributable to selection bias, that is, ECT not being offered to patients in poorer physical health who were more likely to die. However, Rhee et al. (2021) found that patients who were offered ECT, but had a subtherapeutic course, had a similar survival trajectory to the non-ECT group; they suggest the physical health of ECT recipients may improve along with their mental health.

Our meta-analysis for suicide did not show differences in a consistent direction across all studies. There was considerable clinical heterogeneity, and the incidence of suicide varied depending on the time point reported, psychiatric diagnoses studied, and degree of symptom severity. Munk-Olsen et al. (2007) reported patients who had received ECT within the past 7 days and over 4 weeks ago had an increased risk of suicide compared to patients who received no ECT treatment. This was likely affected by selection bias, and the study was not limited to patients with depression: it also included patients with psychotic illnesses. One study of patients with depression also found an increased risk of suicide, but included outpatients (Jørgensen et al., 2020). The study also reported that the HR for suicide was lower in those with psychotic depression compared to mild depression, suggesting the results for severe depression were less affected by residual confounding. Three published studies found ECT was associated with a short-lived protective effect on suicide at 3 months: Rhee et al. (2021), Rönnqvist et al. (2021), and Kaster et al. (2022). The latter two studies used propensity score matching to specifically address confounding by indication, which is a common limitation of the literature on this topic. Of two studies investigating suicide outcomes that were limited to patients with at least moderate depression, one reported a lower risk of suicide in the ECT group (Rönnqvist et al., 2021) and the other reported no increased risk of suicidal behavior (Hedna et al., 2024).

It is possible that ECT has no effect on suicide mortality. However, the absence of a consistent difference in suicide mortality across studies may be explained by the clinical heterogeneity described above. Although the results were highly heterogeneous, studies that restricted the study population to patients with a higher severity of depression and accounted for confounding by indication through a wide range of covariates were more likely to show a reduction in suicide mortality. This interpretation would be in keeping with a recent systematic review and meta-analysis investigating the effect of ECT on mortality in individuals diagnosed with depression (Odermatt et al., 2025). They found a reduction in suicide mortality in this subgroup and, consistent with our findings, a reduction in all-cause mortality in patients treated with ECT.

### Clinical and research implications

Clinical guidelines should be revised to be more realistic about whether ECT reduces suicide risk (and instead specifically suicidal ideation/attempt and overall mortality). Any future RCTs should use longer follow-up periods. A more detailed analysis of the impact of ECT would be facilitated by reporting a wider range of suicide-related outcomes (including suicidal ideation, planning, and attempts). Further studies investigating the risk of suicide are needed, which carefully control for confounding by indication and compare less clinically heterogeneous populations. Further investigation of potential dose–response relationships on these outcomes would be an important research question to address in future studies.

### Strengths and limitations

Strengths of this study include preregistration of the protocol, use of comprehensive search criteria, independent screening, risk of bias rating by clinicians, and meta-analysis. Limitations included amendments due to unanticipated decisions about eligibility arising during screening and the use of a single rater for much of the study screening, data extraction, and risk of bias assessments, which could have contributed to the risk of error or bias. Restricting our search to English and excluding grey literature may have excluded some relevant literature. For self-harm, we were unable to meta-analyze the results, because there were only three studies, which used different self-harm definitions. We acknowledge the absence of lived experience perspectives, which are important for contextualizing results.

## Conclusion

This study found that when prescribed for appropriate indications, ECT is a safe treatment associated with a reduced incidence of mortality, particularly for patients with more severe depression and for older adults. Meta-analysis for suicide did not show a difference in a consistent direction. It is possible that ECT has no effect on suicide mortality. Our included studies that investigated patients with depression of at least moderate severity showed either decreased risk or no increased risk. These findings should be interpreted in the context that the literature on associations between suicidality and ECT is complicated by high heterogeneity in study design, clinical populations studied, follow-up duration, and outcome measures reported.

## Supporting information

Naismith et al. supplementary material Naismith et al. supplementary material

## Supplementary material

The supplementary material for this article can be http://doi.org/10.1017/S0033291725102183.
