# Supplementary material for: Systematic review and meta-analysis of the effectiveness of ECT in reducing suicidal ideation, self-harm, suicide, and mortality
Source: Psychol Med. 2025 Oct 30;55:e328. doi: 10.1017/S0033291725102183 (PMC13054903; doi:10.1017/S0033291725102183)
Supplement: Naismith et al. supplementary material [file S0033291725102183sup001.docx]

Supplementary materials

[Supplementary methods: search strategy 2](#_Toc207372617)

[Supplementary results 4](#_Toc207372618)

[Supplementary figure 1: funnel plot of all-cause mortality 5](#_Toc207372619)

[Supplementary figure 2: funnel plot of non-suicide mortality 6](#_Toc207372620)

[Supplementary figure 3: funnel plot of suicide mortality 7](#_Toc207372621)

[References 8](#_Toc207372622)

## Supplementary methods: search strategy

### Ovid Medline

Exp Electroconvulsive Therapy/

(ECT or Electroshock or Shock?therapy or Electroconvulsive therapy or Convulsive?therapy).ti,ab,kf

self-injurious behavior/ or exp self mutilation/ or suicide/ or exp suicidal ideation/ or exp suicide, attempted/ or exp suicide, completed/

(Self?cut* or Self?mutilat* or Self?poison* or Self?harm* or (Attempt* adj3 suicid) or Suicid or Parasuicid* or Self?injur* or SITB or Self?burn* or deliberate self-harm* or DSH or non?suicidal self?injury or NSSI or non?suicid* or auto?mutilat* or self?destruct* or self?immolat* or self?inflict* or self?injur* or cutt).ti,ab,kf

exp Mortality/

(Fatal or Death or Mortal* or Side?effect or Outcom*).ti,ab,kf

(1 or 2) and (3 or 4 or 5 or 6)

Limit 7 to English

### Ovid EMBASE

exp electroconvulsive therapy/

(ECT or Electroshock or Shock?therapy or Electroconvulsive therapy or Convulsive?therapy).ti,ab,kf

exp suicide/ or exp suicide attempt/ or exp automutilation/

(Self?cut* or Self?mutilat* or Self?poison* or Self?harm* or (Attempt* adj3 suicid) or Suicid or Parasuicid* or Self?injur* or SITB or Self?burn* or deliberate self-harm* or DSH or non?suicidal self?injury or NSSI or non?suicid* or auto?mutilat* or self?destruct* or self?immolat* or self?inflict* or self?injur* or cutt).ti,ab,kf exp mortality/ (Fatal or Death or Mortal* or Side?effect or Outcom*).ti,ab,kf

(1 or 2) and (3 or 4 or 5 or 6)

Limit 7 to English

### Ovid PsycINFO

exp electroconvulsive shock/

(ECT or Electroshock or Shock?therapy or Electroconvulsive therapy or Convulsive?therapy).ti,ab,id

exp self-destructive behavior/ or exp Suicidal Ideation/

(Self?cut* or Self?mutilat* or Self?poison* or Self?harm* or (Attempt* adj3 suicid) or Suicid or Parasuicid* or Self?injur* or SITB or Self?burn* or deliberate self-harm* or DSH or non?suicidal self?injury or NSSI or non?suicid* or auto?mutilat* or self?destruct* or self?immolat* or self?inflict* or self?injur* or cutt).ti,ab,id

exp “death and dying”/

(Fatal or Death or Mortal* or Side?effect or Outcom*).ti,ab,id (1 or 2) and (3 or 4 or 5 or 6)

Limit 7 to English

### Citation searching

Any records not already identified through these database searches but referenced in three previous systematic reviews (Chen et al., 2021; Kucuker et al., 2021; Odermatt et al., 2025) were also included. Chen et al. (2021) and Kucuker et al. (2021) were in our pre-registered protocol. Odermatt et al. (2025) was added following peer reviewer feedback

## Supplementary results

### Change in the suicidality domain of psychological rating scales

The Hamilton Depression Rating Scale (HDRS) captures “hierarchically” feeling that life is not worth living, wishing one were dead or any thoughts of possible death to self; ideas or gestures of suicide; and suicide attempts (Hamilton, 1960). The Beck Depression Inventory (BDI) captures suicidal thoughts (Beck, Ward, Mendelson, Mock, & Erbaugh, 1961). The Columbia-Suicide Severity Rating Scale (C-SSRS) includes both suicidal ideation and suicide attempt (Posner et al., 2011).

Keshtkar, Ghanizadeh, & Firoozabadi (2011) used both the HDRS and the BDI. Lambourn & Gill (1978) used the HDRS suicidality domain. Wang et al. (2024) used the C-SSRS to define suicidal ideation and attempt. The cut-off score on the C-SSRS to define presence or absence of suicidal ideation at the point of discharge is not detailed.

### Suicide and other causes of mortality

In an additional analysis, Kaster et al. (2022) found no association between the number of inpatient ECT treatments and suicide mortality cause-specific hazard ratio 0·99 (95% CI 0·94–1·05), but this was not part of their primary or secondary analyses. This was highlighted as unexpected and as an area requiring further research.

## Supplementary figure 1: funnel plot of all-cause mortality


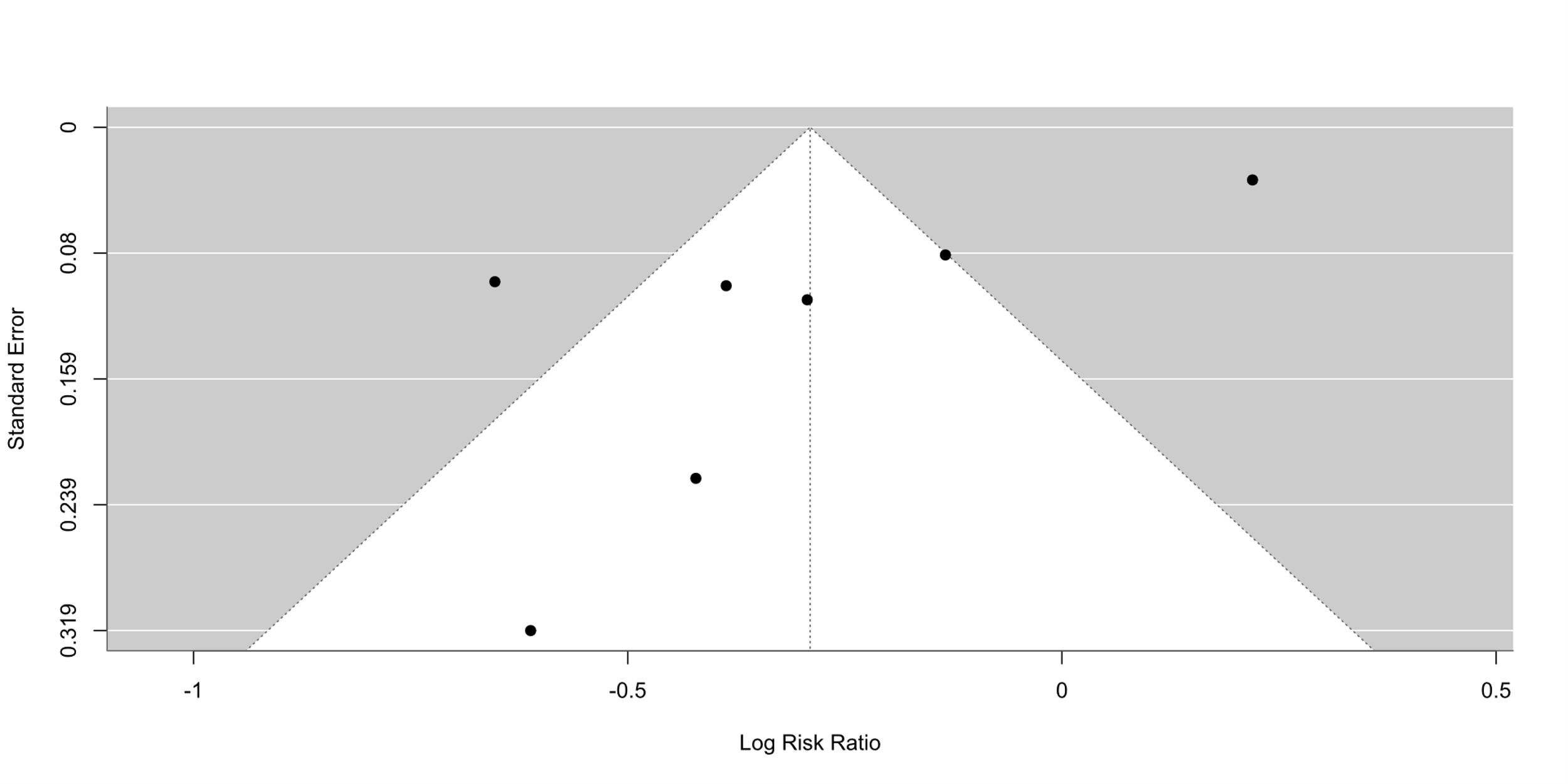


## Supplementary figure 2: funnel plot of non-suicide mortality


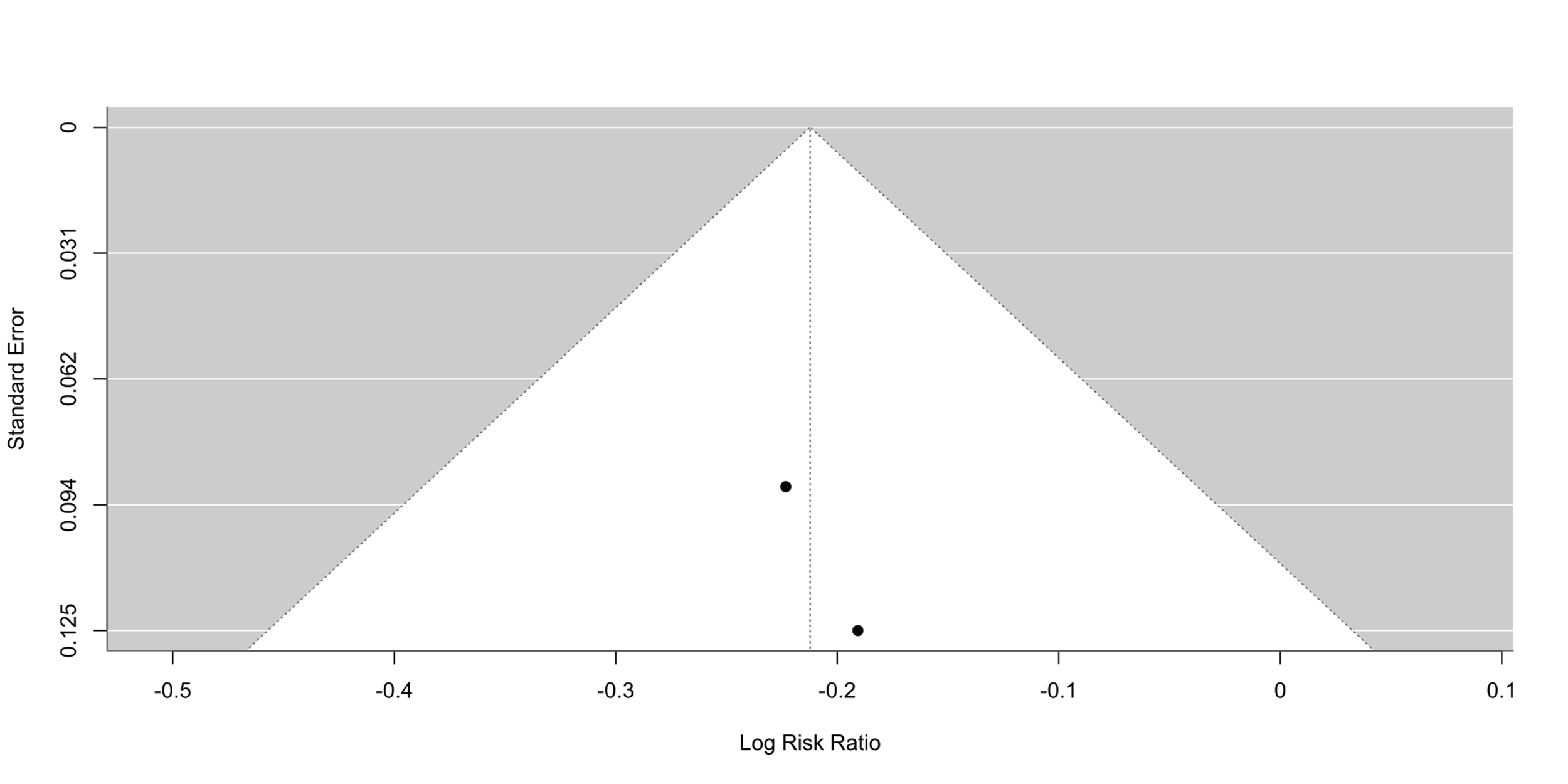


## Supplementary figure 3: funnel plot of suicide mortality


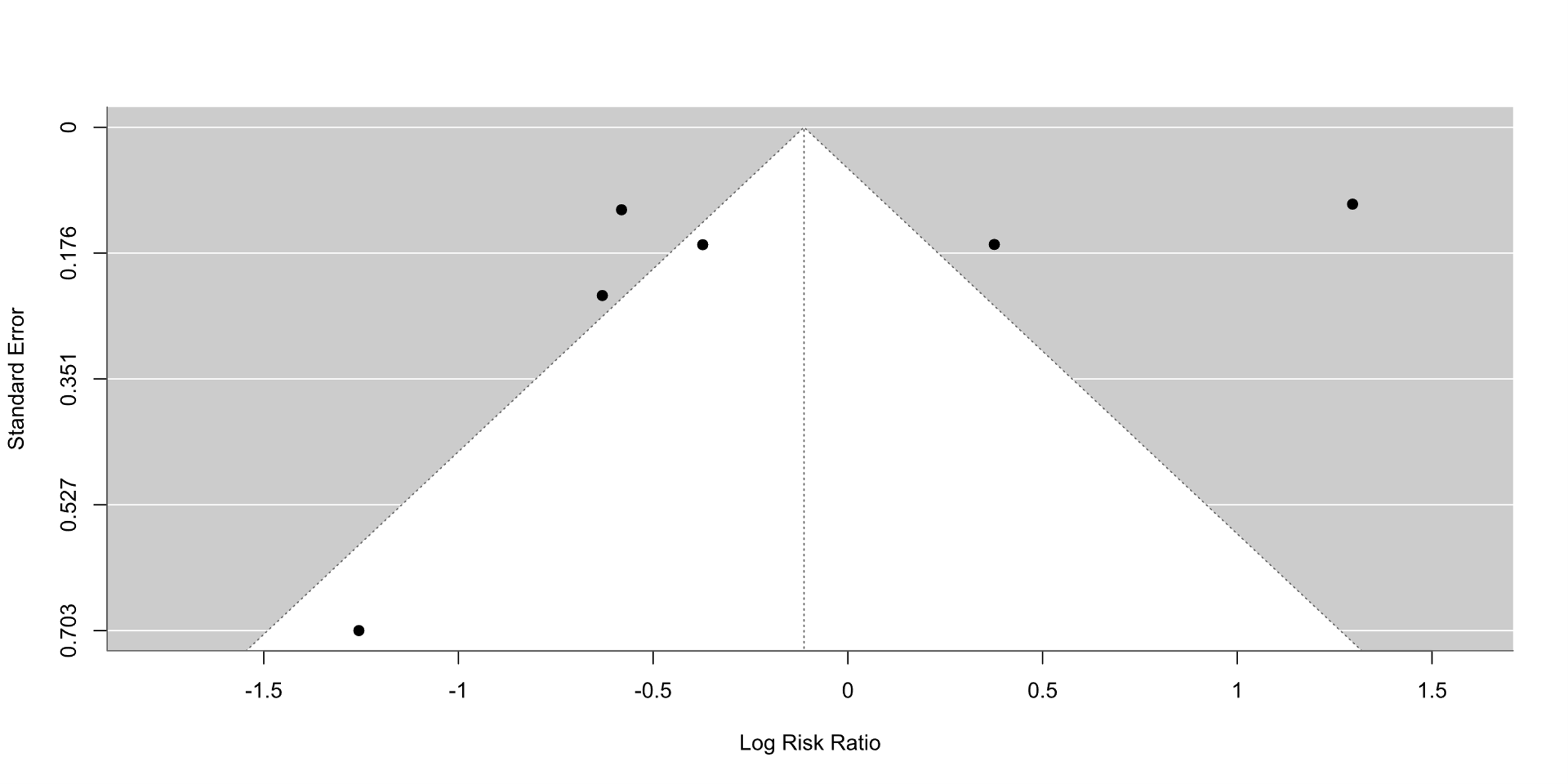


## References

Beck, A. T., Ward, C. H., Mendelson, M., Mock, J., & Erbaugh, J. (1961). An Inventory for Measuring Depression. *Archives of General Psychiatry*, *4*(6), 561. doi: 10.1001/archpsyc.1961.01710120031004

Chen, Y., Magnin, C., Brunelin, J., Leaune, E., Fang, Y., & Poulet, E. (2021). Can seizure therapies and noninvasive brain stimulations prevent suicidality? A systematic review. *Brain and Behavior*, *11*(5), e02144. doi: 10.1002/brb3.2144

Hamilton, M. (1960). A Rating Scale for Depression. *Journal of Neurology, Neurosurgery & Psychiatry*, *23*(1), 56–62. doi: 10.1136/jnnp.23.1.56

Kaster, T. S., Blumberger, D. M., Gomes, T., Sutradhar, R., Wijeysundera, D. N., & Vigod, S. N. (2022). Risk of suicide death following electroconvulsive therapy treatment for depression: A propensity score-weighted, retrospective cohort study in Canada. *The Lancet Psychiatry*, *9*(6), 435–446. doi: 10.1016/S2215-0366(22)00077-3

Keshtkar, M., Ghanizadeh, A., & Firoozabadi, A. (2011). Repetitive Transcranial Magnetic Stimulation Versus Electroconvulsive Therapy for the Treatment of Major Depressive Disorder, A Randomized Controlled Clinical Trial. *The Journal of ECT*, *27*(4), 310–314. doi: 10.1097/YCT.0b013e318221b31c

Kucuker, M. U., Almorsy, A. G., Sonmez, A. I., Ligezka, A. N., Camsari, D. D., Lewis, C. P., & Croarkin, P. E. (2021). A Systematic Review of Neuromodulation Treatment Effects on Suicidality. *Frontiers in Human Neuroscience*, *15*, 305. doi: 10.3389/fnhum.2021.660926

Lambourn, J., & Gill, D. (1978). A Controlled Comparison of Simulated and Real ECT. *British Journal of Psychiatry*, *133*(6), 514–519. doi: 10.1192/bjp.133.6.514

Odermatt, J., Sarlon, J., Schaefer, N., Ulrich, S., Ridder, M., Schneider, E., … Brühl, A. B. (2025). Electroconvulsive therapy reduces suicidality and all-cause mortality in refractory depression: A systematic review and meta-analysis of neurostimulation studies. *Neuroscience Applied*, *4*, 105520. doi: 10.1016/j.nsa.2025.105520

Posner, K., Brown, G. K., Stanley, B., Brent, D. A., Yershova, K. V., Oquendo, M. A., … Mann, J. J. (2011). The Columbia–Suicide Severity Rating Scale: Initial Validity and Internal Consistency Findings From Three Multisite Studies With Adolescents and Adults. *American Journal of Psychiatry*, *168*(12), 1266–1277. doi: 10.1176/appi.ajp.2011.10111704

Wang, X., Ren, H., Zhang, Z., Zhong, X., Luo, Q., Huang, Y., & Qiu, H. (2024). The efficacy of electroconvulsive therapy in adolescent major depressive disorder with suicidal ideation: A propensity score-matched, retrospective cohort study. *Asian Journal of Psychiatry*, *95*, 103998. doi: 10.1016/j.ajp.2024.103998
